# Transforming presumptive forensic testing: *in situ* identification and age estimation of human bodily fluids†
†Electronic supplementary information (ESI) available. See DOI: 10.1039/c8sc04133d


**DOI:** 10.1039/c8sc04133d

**Published:** 2018-11-07

**Authors:** Stephanie Rankin-Turner, Matthew A. Turner, Paul F. Kelly, Roberto S. P. King, James C. Reynolds

**Affiliations:** a Department of Chemistry , Loughborough University , Loughborough , LE11 3TU , UK . Email: J.C.Reynolds@lboro.ac.uk; b Foster + Freeman Ltd , Vale Park, Evesham , Worcestershire WR11 1TD , UK

## Abstract

The ability to achieve rapid, *in situ* identification and age estimation of human bodily fluids can provide valuable information during the investigation of a crime. A novel direct analysis method now permits the rapid *in situ* identification and age estimation of human bodily fluids for forensic analysis at crime scenes. A thermal desorption surface sampling probe was developed and coupled with a compact mass spectrometer for the direct analysis of volatile organic compound (VOC) profiles of human bodily fluids within two months and in different environmental conditions, without the need for prior sample preparation. The method is not only capable of identifying bodily fluids and discriminating against common interferent species, but also differentiating between bodily fluid stains of different ages over a time period of two months. This demonstrates the potential for rapid *in situ* identification and age estimation of bodily fluids without the need for contaminative presumptive tests or time-consuming sample preparation.

## Abstract 2

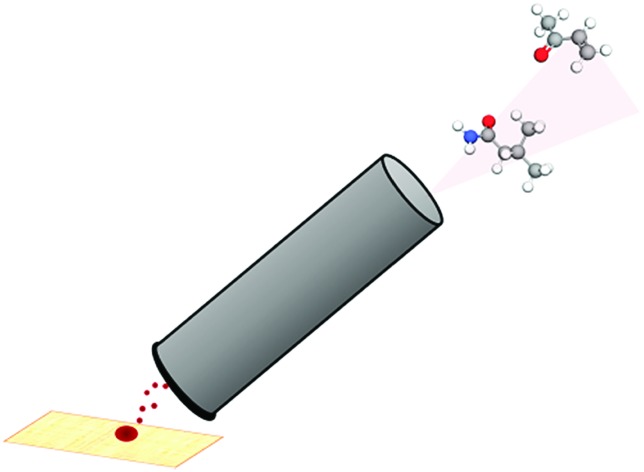
A new method utilising an *in situ* sampling probe coupled with mass spectrometry for rapid identification and age estimation of biofluids.

## Introduction

Biological samples recovered from crime scenes can play an essential role in a criminal investigation, providing vital information as to the sequence of events and to the involvement of particular individuals. It is, therefore, crucial that suspected biofluids are promptly detected and identified; unfortunately, current bodily fluid identification and analysis procedures are far from ideal. Methods for the presumptive identification of bodily fluids utilise chemical tests which involve the addition of a particular reagent to the suspected biofluid, producing a chemiluminescence or colour change often driven by enzyme activity in the presence of a particular bodily fluid.^1^ These presumptive tests typically lack specificity, resulting in the possibility of false positive results. Conducting laboratory-based confirmatory analysis and DNA profiling can take days to complete,^2^ slowing the investigation. In addition, the need to perform DNA profiling of bodily fluids necessitates the preservation of the sample, therefore contamination of samples by presumptive chemical tests or sample destruction by confirmatory analysis is not ideal. In recent years there has been a push towards the development of technology that can be deployed at a crime scene.^3^

Mass spectrometry (MS) has been utilised extensively for the metabolic profiling of human bodily fluids. A substantial number of studies have been conducted with the aim of characterising the entire metabolome of human bodily fluids, including saliva,^4^ urine^5^ and serum,^6^ using mass spectrometry and other analytical methods. Furthermore, the success of MS techniques for the analysis of biofluids in a forensic context have been demonstrated on numerous occasions, both for the characterisation of bodily fluids,^7^,^8^ and for the differentiation of bodily fluids from different donors.^9^–^12^

All of these studies, however, necessitated some form of sample preparation and chromatographic separation prior to analysis, in some cases taking hours to analyse a single sample. The development of a range of techniques known as ambient ionisation mass spectrometry has introduced the prospect of conducting sample analysis without the need for preparation and pre-separation, opening up the possibility that *in situ* analysis of samples in their native state may be performed. The advent of ambient ionisation was largely triggered by the introduction of desorption electrospray ionisation (DESI) in 2004 13 and direct analysis in real time (DART) in 2005,^14^ which demonstrated the possibility of ionisation and sampling of analytes directly from the surface of a sample. Since then, over 80 variations of ambient ionisation MS have been developed.^15^

Despite these developments, to date there has been limited success in the development of an *in situ* method for the forensic identification of human bodily fluids. Although Takáts *et al.* developed rapid evaporative ionisation mass spectrometry (REIMS) for the direct *in situ* analysis of biological tissue for medical applications,^16^ this technique would not be suited to the analysis of forensic bodily fluid samples due to its destructive nature. In addition, many researchers have aimed to develop analytical methods for the age estimation of bodily fluids,^17^–^21^ however there are still no accepted, established methods. Developing a non-destructive method of *in situ* sampling of suspected bodily fluids, and combining this with analysis using an ambient ionisation mass spectrometry technique, could allow for the discrimination and age estimation of bodily fluids without the need for contaminating chemical reagents or time-consuming sample preparation steps. This in turn would accelerate the process of identifying suspected biofluids for investigation, reducing the number of samples being sent for confirmatory DNA analysis and the cost to the investigating authorities. Here we report the development of a method that achieves this objective.

## Experimental

**Fig. 1:**
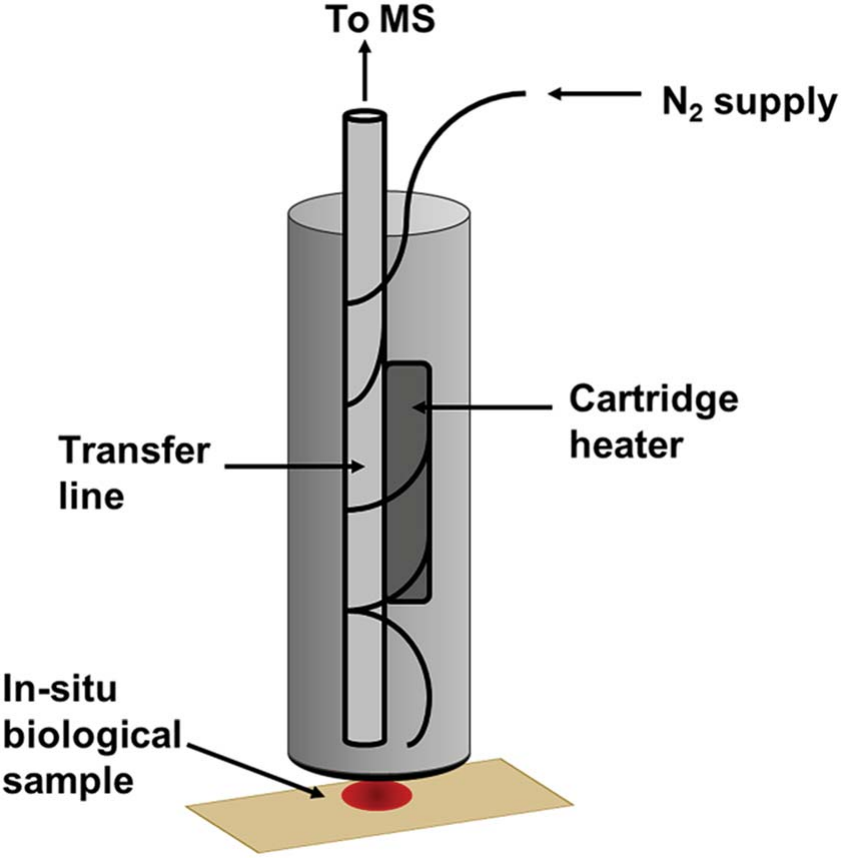
Desorption off surface (DOS) probe.

Thermal desorption (TD) has been extensively utilised with mass spectrometry. In particular, TD has been frequently coupled with atmospheric pressure chemical ionisation (TD-APCI-MS) in the analysis of drugs^22^ and explosives.^23^ This has demonstrated the possibility of releasing VOCs from a sample for immediate analysis. The Desorption Off Surface (DOS) probe (Fig. 1) is a handheld device incorporating a gas line to direct a stream of heated N_2_ gas towards the sample surface and a transfer line to transport thermally desorbed VOCs to the analyser. The heated gas line is positioned approximately 1 mm above the sample surface. An insulated outer covering protects the operator from heat produced by the probe and an O-ring positioned at the base of the probe ensures a tight seal between the probe and the sampling surface to avoid loss of VOCs. This maintains a uniform distance between the transfer line and the sample whilst not making physical contact with the sample itself. The sampling probe was set to produce a sample surface temperature of ∼60 °C, sufficient to achieve thermal desorption of VOCs whilst preserving the sample itself, as DNA should not degrade at this temperature.^24^ This operating temperature also effectively enables the discrimination of VOCs from involatile interferences such as fats, sugars and proteins and many semivolatile organic compounds (SVOCs), simplifying the spectra obtained. The sampling probe outlet was interfaced with the transfer line of an Advion Expression compact mass spectrometer (CMS) with a volatile atmospheric pressure chemical ionisation (vAPCI) source, operating in positive ionisation mode (full experimental details in ESI†). The 1/8′′ i.d silcosteel transfer line was coupled with a Venturi jet pump, with a suction of 0.6 L min^–1^ and heated to 100 °C. The N_2_ supply entering the sampling probe was first fed through a glass vial containing a deuterated acetone permeation source, providing a constant introduction of acetone-d_6_ as an internal standard throughout sample analysis, with a flow of 0.5 L min^–1^ (Fig. S1†).

As part of the initial screening study, human blood, saliva and urine samples were collected, pooled and immediately stored at –80 °C in order to preserve VOCs until analysis (stored for approximately 4 weeks). Interferents were stored at room temperature. This study was undertaken in accordance with the ethical standards of the university and the Declaration of Helsinki. Samples were collected from volunteer participants who gave informed consent (Table S1†). Interferents are materials that may be visually similar to a bodily fluid or may react in a similar manner during the application of a chemical presumptive test. Interferents to be analysed in this study included the following commercially available food and cosmetic products: body lotion, coffee, Durex Play lubricant, soy sauce, Sudocrem, sun lotion, tea, and tomato sauce. 10 μl of bodily fluid or interferent was applied to Fisherbrand grade 601 filter paper (Fisher Scientific, Loughborough, UK) and either analysed immediately or stored for ageing purposes. Multiple replicates were prepared and new body fluid spots analysed at each time point to ensure sample ageing was not affected by the application of the DOS probe. Aged samples were stored under ambient conditions without controlled temperature or humidity. Furthermore, additional blood aliquots were stored under light and dark conditions for the purpose of investigating the effects of light exposure on chemical ageing of body fluids (with “light” conditions being exposure to the normal light/dark cycle and “dark” conditions being the complete isolation from any light source). During sampling, the DOS probe was directly applied to the biological fluid, allowing the heated N_2_ gas to thermally desorb volatile compounds from the sample, which were then transported to the mass spectrometer for analysis. Sampling time was approximately 2 minutes per sample (1 minute blank filter paper analysis followed by 1 minute sample analysis), with real-time analysis displayed on-screen. The subtraction of the blank from samples enables background interferents to be removed prior to multivariate analysis.

Accurate masses of compounds of interest were obtained by coupling the DOS probe with an Orbitrap mass spectrometer using an extractive electrospray ionisation (EESI) interface. The outlet of the thermal desorption probe was coupled with a Venturi pump to direct the outlet flow into the plume of an electrospray situated at the atmospheric inlet of the mass spectrometer. The pump outlet was positioned opposite the inlet and the electrospray was positioned perpendicular to the inlet. Unaged and aged bodily fluid samples were then analysed as previously described. The mechanism of extractive electrospray ionisation is described in more detail elsewhere,^25^,^26^ and has been effectively utilised with neutral desorption for the analysis of food products^27^ and thermal desorption in urine analysis.^28^ Extracted data were subjected to background subtraction, corrected to the internal standard, and imported into SIMCA P+ software (version 14, Umetrics, Sweden) for multivariate analysis by principal component analysis (PCA) and partial least squares discriminant analysis (PLS-DA). PLS-DA was initially used to identify discriminating variables and construct a predictive model, after which unsupervised PCA models were constructed using these selected variables. Class ellipses were manually added to show relative symbol boundaries for each class in the PCA models.

## Results and discussion

**Fig. 2:**
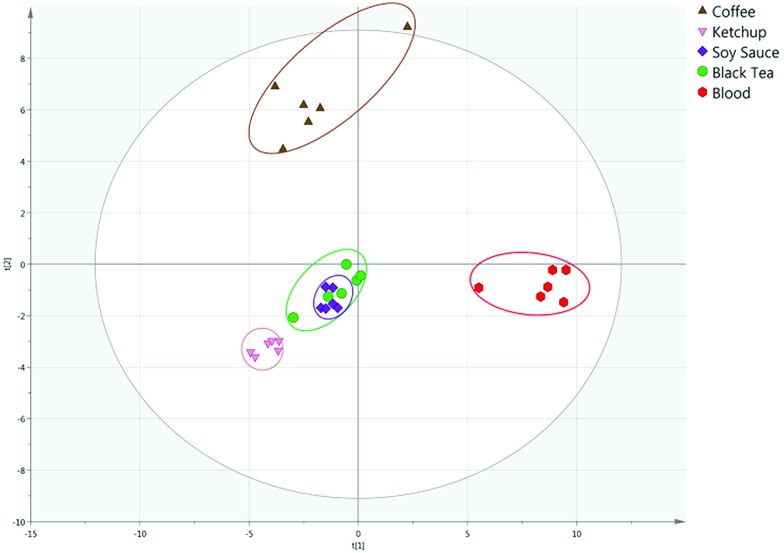
PCA score plot of unaged blood and possible interferents.

The PCA model could readily separate blood, saliva and urine samples, indicating the VOC profiles of the bodily fluids are sufficiently distinct to differentiate between and allow identification of different bodily fluids. The strength of the model can be indicated by the *R*^2^ and *Q*^2^ values, which describe how well the model fits the data and the predictability of the model respectively. Unaged blood, saliva and urine analysed were differentiated with PC1 and PC2 explaining 66.9 and 13.3% of variance, with an *R*^2^ of 0.855 and a *Q*^2^ of 0.714 (Fig. S3†). Furthermore, the model clearly separated blood, saliva and urine from potential interferences. Unaged blood was separated from visually similar interferents, with the first two principal components explaining 40.8 and 23.3% of the total variance, with an *R*^2^ of 0.821 and a *Q*^2^ of 0.632 (Fig. 2).

Similarly, saliva and urine were also differentiated from possible interferences. Saliva was clearly separated from interferents with PC1 and PC2 explaining 40.6 and 20.5% of variance respectively, with an *R*^2^ of 0.896 and a *Q*^2^ of 0.635 (Fig. S4†). Urine was differentiated from interferents with 34.4 and 18.4% of variance explained, and an *R*^2^ or 0.771 and a *Q*^2^ of 0.608 (Fig. S5†). This demonstrates the potential of the technique to distinguish blood, saliva and urine from innocuous substances that may be mistaken for the bodily fluid under visual, or presumptive chemical, inspection at a crime scene.

**Fig. 3:**
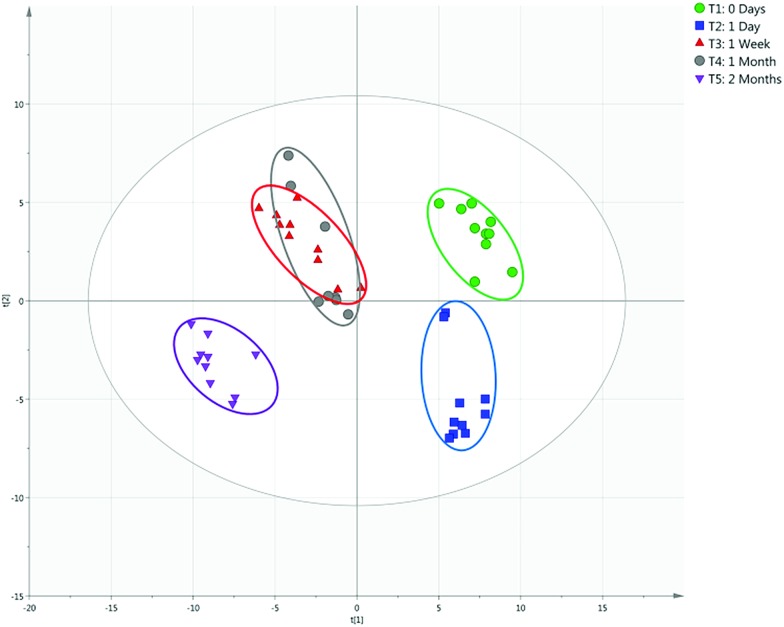
PCA score plot of blood aged 0 days to 2 months.

In the context of a criminal investigation, solely establishing the identity of a suspected bodily fluid may not provide sufficient information. For instance, the confirmation of the presence of blood alone may not be pertinent to the incident under investigation without knowing for how long that bloodstain has been present. Accordingly, blood, saliva and urine samples were applied to filter paper, stored under ambient conditions and analysed at multiple time-points over a period of two months. 10 biofluid samples were analysed at each time point. PCA models were developed that were capable of differentiating between bodily fluids of different ages, with some overlap observed between certain time-points. Aged blood samples were differentiated with PC1 explaining 38.4% of variation and PC2 explaining 15.5% of the variation, with an *R*^2^ value of 0.615 and a *Q*^2^ value of 0.462 (Fig. 3). Day 0 and 1 day old samples are distinguishable largely due to the greater intensity of compounds, which is to be anticipated as the concentration of VOCs present in the samples will decrease over time as they are lost to the external environment. Blood at the 1 week and 1 month time-points exhibited more overlap in the PCA model, likely due to the variation in response at these timepoints resulting in increased overlap. By including only 1 week and 1 month samples in the model, separation is observed between the two classes (Fig. S9†). However the samples stored for 2 months could be clearly differentiated. Fig. S10† demonstrates the mass spectral differences between the first and final timepoints. PCA score plots of aged saliva and urine can be found in Fig. S11–S13,† along with blood, saliva and urine at the 2 months time point (Fig. S14†).

**Fig. 4:**
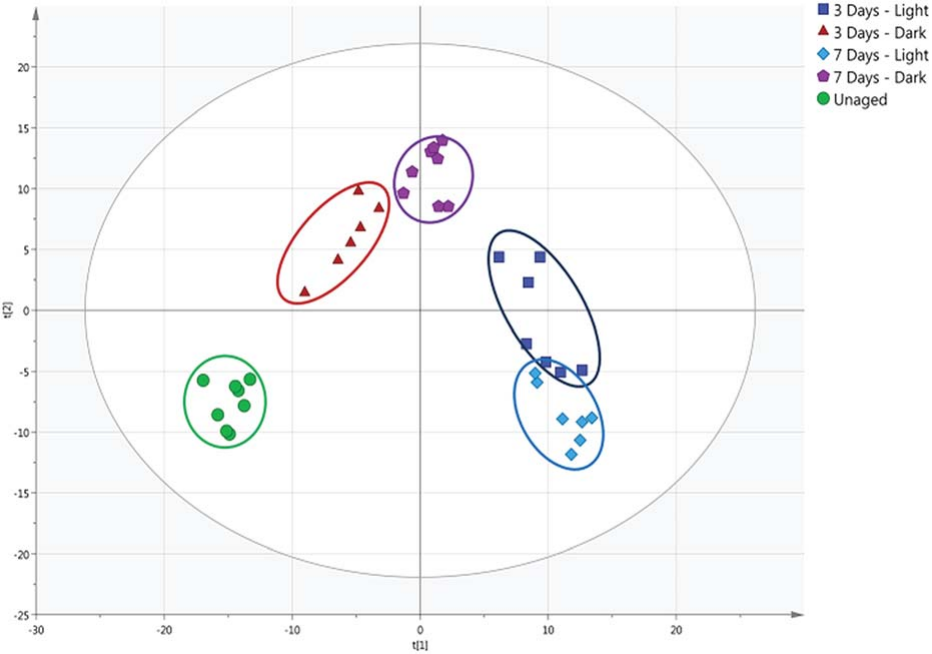
PCA score plot of unaged and aged blood stored under light and dark conditions.

Fig. 4 demonstrates the importance of understanding the effects of environmental conditions on the ageing of bodily fluids. A PCA plot of blood samples stored in light and dark conditions over a period of seven days (*R*^2^ of 0.590 and *Q*^2^ of 0.490) highlights the chemical differences occurring, with an evident separation observed between light-aged and dark-aged samples. It has been previously suggested that the ageing of blood may be affected by lighting conditions,^17^ most likely due to the presence of photosensitive compounds whose degradation is accelerated by exposure to UV light. Environmental temperature may also have contributed to these differences, with samples exposed to sunlight likely to have experienced slightly higher temperatures than those stored in darkness.

Although the majority of compounds detected decreased in concentration over time, a number of compounds were found to increase in concentration with the age of the bodily fluid. These were investigated using the high resolution EESI Thermo Orbitrap method, enabling molecular formulae to be obtained from accurate mass analyses. These were compared with the Human Metabolome Database^29^ and existing literature exploring the presence of volatile organic compounds in bodily fluids, and putative identifications made.

**Fig. 5:**
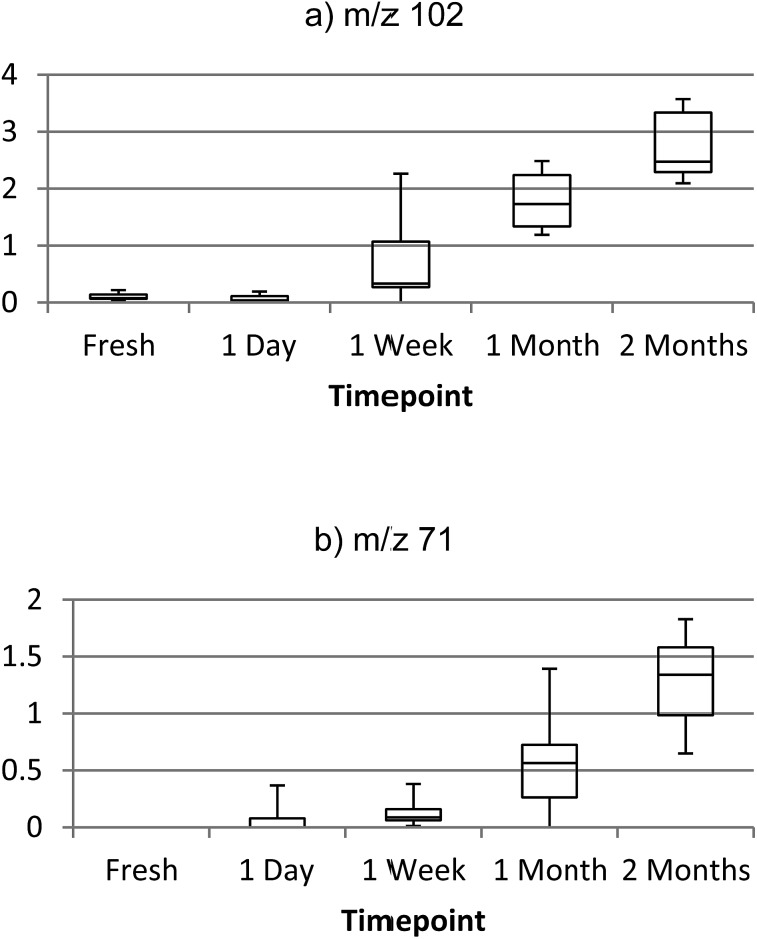
Box plots of ion intensity increasing with age of bloodstain sample, (a) *m*/*z* 102 (3-methylbutanamide) and (b) *m*/*z* 71 (3-buten-2-one).

2-Pyrrolidinone, 3-methylbutanamide, 4-methyl-4-hexen-3-one, 6-methyl-5-hepten-2-one, phenylacetone and 2-pentylfuran were amongst the compounds found to be increasing in concentration as the blood sample aged, and all of these species have been previously detected in aged blood samples elsewhere,^30^,^31^ supporting the putative annotations in Table 1. However analysis by additional analytical methods would be required to confirm these suggestions. This suggests the potential of utilising the presence and concentration of these particular chemical markers to estimate the time since deposition of a bloodstain (see Fig. 5 and S15†).

**Table 1:** A selection of compounds that increase in concentration with the age of the bloodstain

Observed mass	Formula	Mass error (ppm)	Potential compound
71.04935	C_4_H_7_O	1.388	3-Buten-2-one
86.06047	C_4_H_8_NO	2.319	2-Pyrrolidinone
102.09146	C_5_H_12_NO	–1.457	3-Methylbutanamide
113.09578	C_7_H_13_O	1.666	4-Methyl-4-hexen-3-one
127.11185	C_8_H_15_O	0.852	6-Methyl-5-hepten-2-one
135.07987	C_9_H_11_O	1.691	Phenylacetone
139.11173	C_9_H_15_O	–0.084	2-Pentylfuran

For instance, concentration of 3-methylbutanamide was found to be significantly different between all timepoints (*p* = <0.05), with the exception of 0 day and 1 day old samples. A series of 3-methylbutanamide standard solutions were analysed to produce a linear calibration (Fig. S16†), with an *R*^2^ of 0.9959 and established a limit of detection for this species of approximately 10 ng on the surface. A number of identified compounds were also found to increase in saliva and urine samples, however this requires further confirmative investigation as there are relatively few published chemical markers for aged urine and saliva samples.

## Conclusions

A direct surface analysis method has been developed for the rapid, *in situ* characterisation of human bodily fluids, also enabling preservation of the sample for subsequent DNA analysis. With the use of a custom-built thermal desorption sampling probe coupled with a compact mass spectrometer, human blood, saliva and urine and common interferents could be directly analysed and identified based on unique VOC profiles. Multivariate analysis allowed for the specific ions unique to the bodily fluids to be identified and targeted for future analyses and potential sensing platforms. The method has the ability to differentiate between bodily fluids of different ages and provides a method for estimation of time since deposition of the stain. This method has demonstrated the possibility of direct analysis for the identification and age estimation of a suspected bodily fluid *in situ*, without the need for the addition of presumptive chemical tests and time-consuming sample preparation steps prior to analysis. In this instance the DOS probe was coupled with an Advion Expression compact mass spectrometer, an instrument intended for easy transportation for on-site use. However the probe could be easily coupled with a range of fully portable instruments, including mass spectrometers and ion mobility spectrometers. Environmental factors, for example light exposure, significantly affect the VOC profile emitted by an aged blood sample. The methodology is capable of distinguishing between samples aged under different environmental conditions, therefore an age estimation model would need to take environmental factors into account. Further work will involve a larger-scale study including the application of the method to additional bodily fluids of forensic interest, such as semen and vaginal secretions. This study will also seek to validate the markers identified here and also further investigate the effects of different environmental conditions (surface type, temperature, humidity), and surface contamination effects. Furthermore, as this proof of principle study does not include external validation of the PCA models constructed, additional validation experiments are required to increase confidence in the models before this methodology can be deployed for use in criminal investigations. However, the work reported here clearly demonstrates the effectiveness of this new approach and its potential to solve vital forensic challenges.

## Conflicts of interest

There are no conflicts to declare.

## Supplementary Material

Supplementary information

## References

[cit1] Virkler K., Lednev I. K. (2009). Forensic Sci. Int..

[cit2] Home Office, National DNA Database Strategy Board Annual Report 2015–2016, 2016.

[cit3] Small Business Research Initiative, Call for the development of novel techniques for gathering forensic evidence, https://sbri.innovateuk.org/competition-display-page/-/asset_publisher/E809e7RZ5ZTz/content/forensics/1524978, (accessed 10 January 2018).

[cit4] Dame Z. T., Aziat F., Mandal R., Krishnamurthy R., Bouatra S., Borzouie S., Guo A. C., Sajed T., Deng L., Lin H., Liu P., Dong E., Wishart D. S. (2015). Metabolomics.

[cit5] Bouatra S., Aziat F., Mandal R., Guo A. C., Wilson M. R., Knox C., Bjorndahl T. C., Krishnamurthy R., Saleem F., Liu P., Dame Z. T., Poelzer J., Huynh J., Yallou F. S., Psychogios N., Dong E., Bogumil R., Roehring C., Wishart D. S. (2013). PLoS One.

[cit6] Psychogios N., Hau D. D., Peng J., Guo A. C., Mandal R., Bouatra S., Sinelnikov I., Krishnamurthy R., Eisner R., Gautam B., Young N., Xia J., Knox C., Dong E., Huang P., Hollander Z., Pedersen T. L., Smith S. R., Bamforth F., Greiner R., McManus B., Newman J. W., Goodfriend T., Wishart D. S. (2011). PLoS One.

[cit7] Kusano M., Mendez E., Furton K. G. (2011). Anal. Bioanal. Chem..

[cit8] Kamanna S., Henry J., Voelcker N. H., Linacre A., Kirkbride K. P. (2016). Int. J. Mass Spectrom..

[cit9] Kusano M., Mendez E., Furton K. G. (2013). J. Forensic Sci..

[cit10] Brown J. S., Prada P. A., Curran A. M., Furton K. G. (2013). Forensic Sci. Int..

[cit11] Colon-Crespo L. J., Herrera-Hernandez D., Holness H., Furton K. G. (2017). Forensic Sci. Int..

[cit12] Penn D. J., Oberzaucher E., Grammer K., Fischer G., Soini H. A., Wiesler D., V Novotny M., Dixon S. J., Xu Y., Brereton R. G. (2007). J. R. Soc., Interface.

[cit13] Takáts Z., Wiseman J. M., Gologan B., Cooks R. G. (2004). Science.

[cit14] Cody R. B., Laramée J. A., Durst H. D. (2005). Anal. Chem..

[cit15] Javanshad R., Venter A. R. (2017). Anal. Methods.

[cit16] Schäfer K.-C., Dénes J., Albrecht K., Szaniszló T., Balog J., Skoumal R., Katona M., Tóth M., Balogh L., Takáts Z. (2009). Angew. Chem., Int. Ed..

[cit17] Bremmer R. H., De Bruin K. G., Van Gemert M. J. C., Van Leeuwen T. G., Aalders M. C. G. (2012). Forensic Sci. Int..

[cit18] Doty K. C., Muro C. K., Lednev I. K. (2017). Forensic Chem..

[cit19] Li B., Beveridge P., O'Hare W. T., Islam M. (2013). Sci. Justice.

[cit20] Agudelo J., Huynh C., Halámek J. (2015). Analyst.

[cit21] Edelman G., Manti V., van Ruth S. M., van Leeuwen T., Aalders M. (2012). Forensic Sci. Int..

[cit22] Sleeman R., Burton I. F. A., Carter J. F., Roberts D. J. (1999). Analyst.

[cit23] Popov I. A., Chen H., Kharybin O. N., Nikolaev E. N., Cooks R. G. (2005). Chem. Commun..

[cit24] Karni M., Zidon D., Polak P., Zalevsky Z., Shefi O. (2013). DNA Cell Biol..

[cit25] Chen H., Venter A., Cooks R. G. (2006). Chem. Commun..

[cit26] Law W. S., Wang R., Hu B., Berchtold C., Meier L., Chen H., Zenobi R. (2010). Anal. Chem..

[cit27] Wu Z., Chingin K., Chen H., Zhu L., Jia B., Zenobi R. (2010). Anal. Bioanal. Chem..

[cit28] Devenport N. A., Blenkhorn D. J., Weston D. J., Reynolds J. C., Creaser C. S. (2014). Anal. Chem..

[cit29] Wishart D. S., Jewison T., Guo A. C., Wilson M., Knox C., Liu Y., Djoumbou Y., Mandal R., Aziat F., Dong E., Bouatra S., Sinelnikov I., Arndt D., Xia J., Liu P., Yallou F., Bjorndahl T., Perez-Pineiro R., Eisner R., Allen F., Neveu V., Greiner R., Scalbert A. (2013). Nucleic Acids Res..

[cit30] Forbes S. L., Rust L. T., Trebilcock K., Perrault K. A., McGrath L. T. (2014). Forensic Sci., Med., Pathol..

[cit31] Rust L. T., Nizio K. D., Forbes S. L. (2016). Anal. Bioanal. Chem..

