# Supplementary material for: Transforming presumptive forensic testing: in situ identification and age estimation of human bodily fluids
Source: Chem Sci. 2018 Nov 7;10(4):1064–9. doi: 10.1039/c8sc04133d (PMC6346410; doi:10.1039/c8sc04133d)
Supplement: Supplementary file 1 [file SC-010-C8SC04133D-s001.pdf]

Electronic Supplementary Material (ESI) for Chemical Science.
This journal is © The Royal Society of Chemistry 2018

# Transforming Presumptive Forensic Testing: In-situ Identification and Age Estimation of Human Bodily Fluids

Stephanie Rankin-Turner, Matthew A. Turner, Paul F. Kelly, Roberto S. P. King, James C. Reynolds

## Supplementary Information

## Experimental Section

Thermal desorption of samples was achieved using a custom-built sampling probe comprised of a 12cm stainless steel tube with an internal diameter of 18mm, enclosing a 3mm i.d stainless steel tube to transfer analytes from the sampling surface to the analyser and a 1.5mm i.d silicosteel gas line to introduce a stream of $N_2$ gas to the sample surface. A cartridge heater affixed to the line heats the gas in order to facilitate desorption of volatile compounds from the sampling surface. Experiments were conducted using an Advion Expression Compact Mass Spectrometer (Advion, UK) fitted with an atmospheric pressure chemical ionisation (APCI) source, operating in positive ionisation mode. The 1/8" i.d silcosteel transfer line was coupled with a Venturi jet pump, with a suction of 0.6 L/min and heated to 100$^{o}$C. Ion source parameters were as follows: capillary temperature 250$^{o}$C, capillary voltage 110 V, source offset voltage 16 V, APCI source gas temperature 350$^{o}$C, and corona discharge voltage 4 μA. Spectra were acquired over a range of 30 to 300 m/z. Compound identification was carried out using an Orbitrap Exactive mass spectrometer (Thermo, Bremen, Germany) with extractive electrospray ionisation. Orbitrap experiments were carried out in positive ionisation mode with caffeine (Alfa Aesar, Heysham, UK) as a lockmass with the following parameters: scan range: 50-300 m/z, spray voltage 3 kV, capillary voltage 30V, tube lens voltage 80 V, and skimmer voltage 20 V.

## Additional Figures

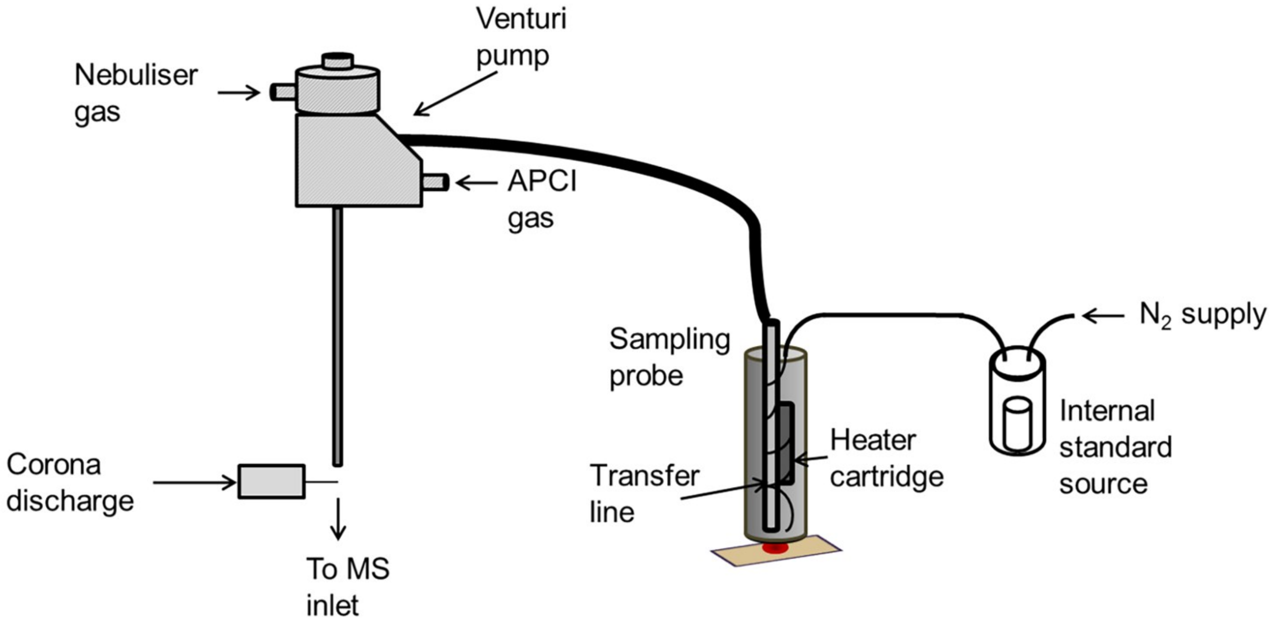


**Figure S1: Full experimental setup schematic.**

This study was conducted in accordance with the Declaration of Helsinki, and samples were collected under the following ethics: R18-P034

| Participant | Sex | Age | Race / Ethnicity |
|---|---|---|---|
| Participant 1* | Male | 40 | Caucasian |
| Participant 2 | Male | 37 | Caucasian |
| Participant 3 | Male | 35 | Black African |
| Participant 4 | Male | 27 | Asian |
| Participant 5 | Female | 28 | Caucasian |
| Participant 6 | Female | 26 | Caucasian |

**Table S1: Donor participant information (blood n=1, saliva n=6, urine n=6, *blood donor).**

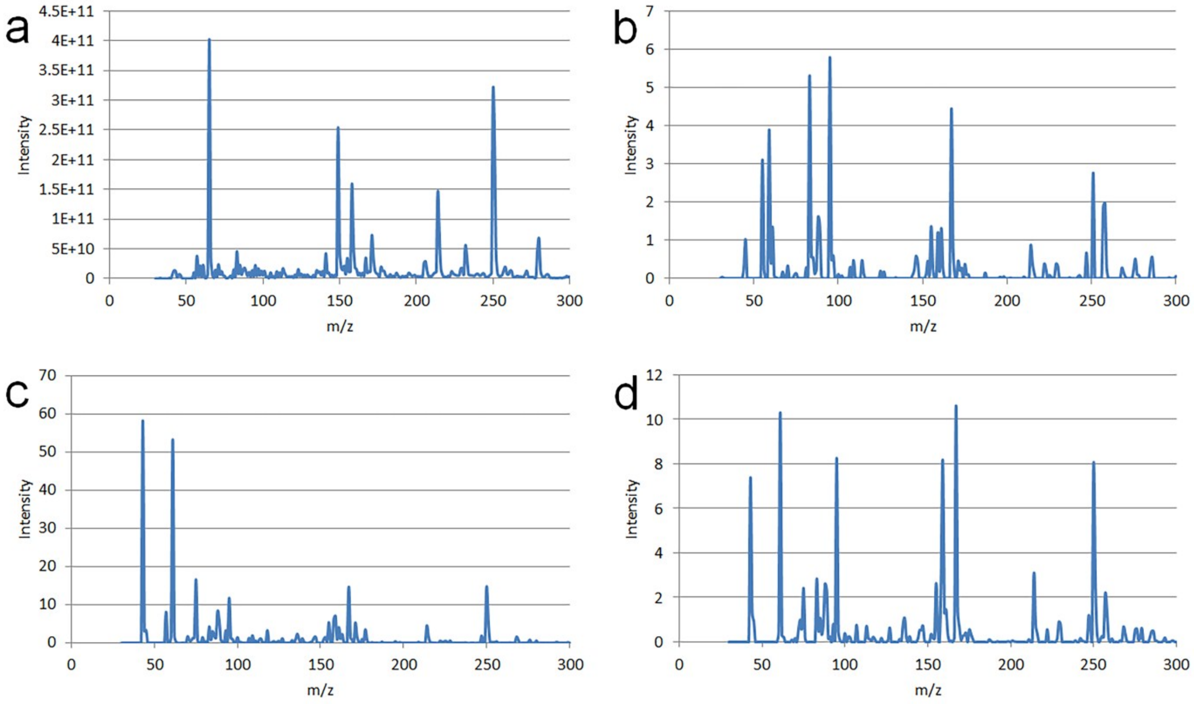


**Figure S2: Mass spectra of blank filter paper (a) and unaged blood (b), saliva (c) and urine (d) corrected against the internal standard.**

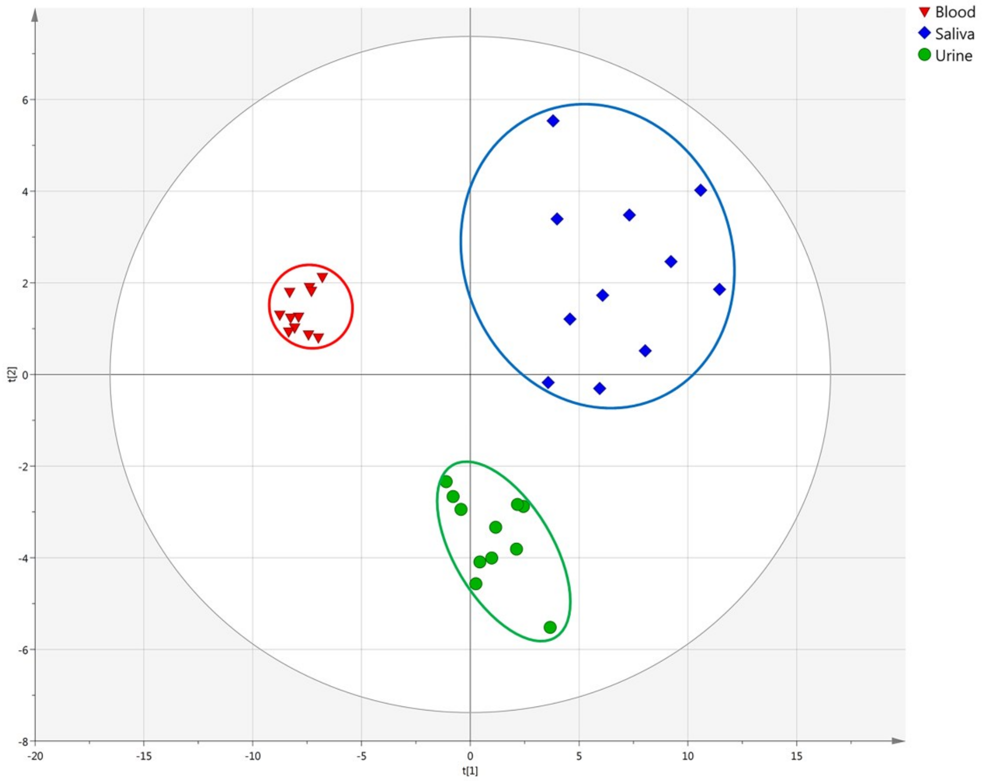


**Figure S3: PCA score plot of unaged blood, saliva and urine.**

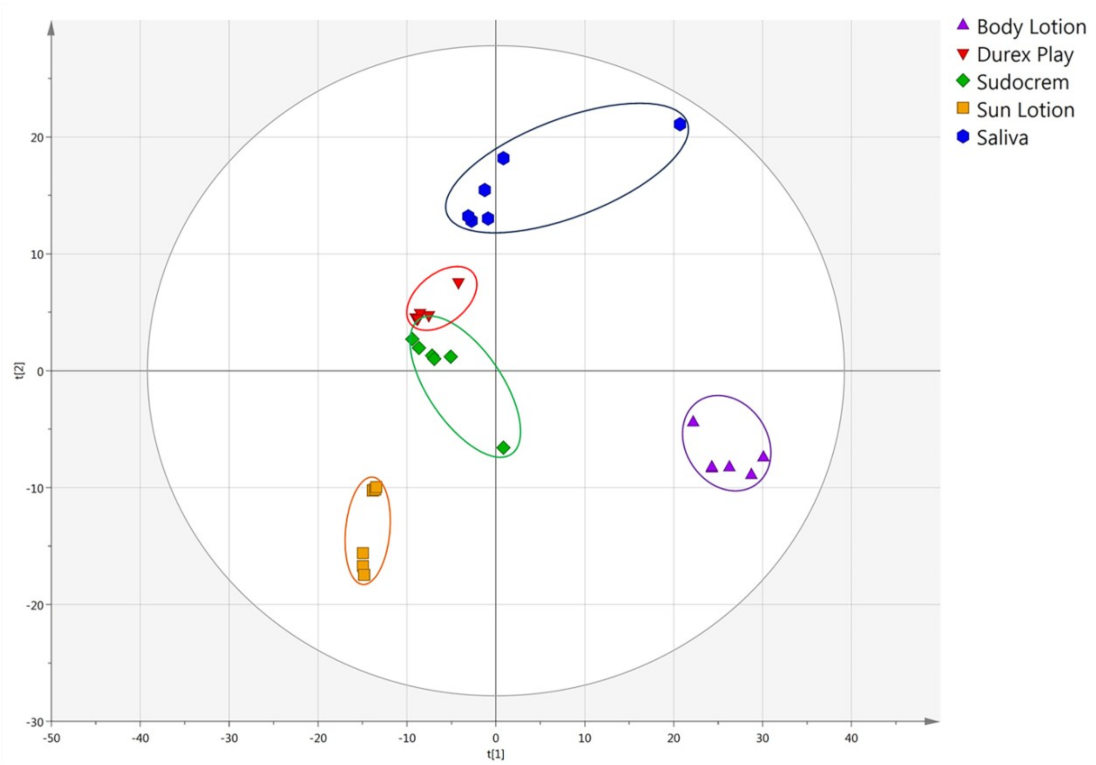


**Figure S4: PCA score plot of saliva and possible interferents.**

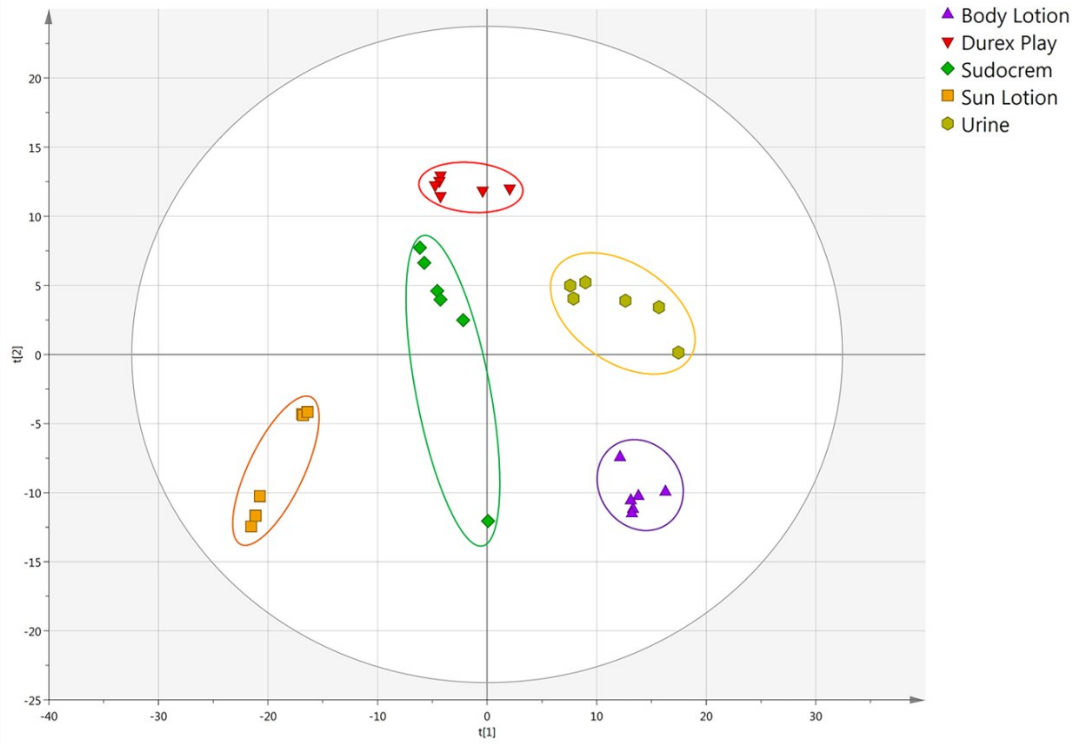


**Figure S5: PCA score plot of urine and possible interferents.**

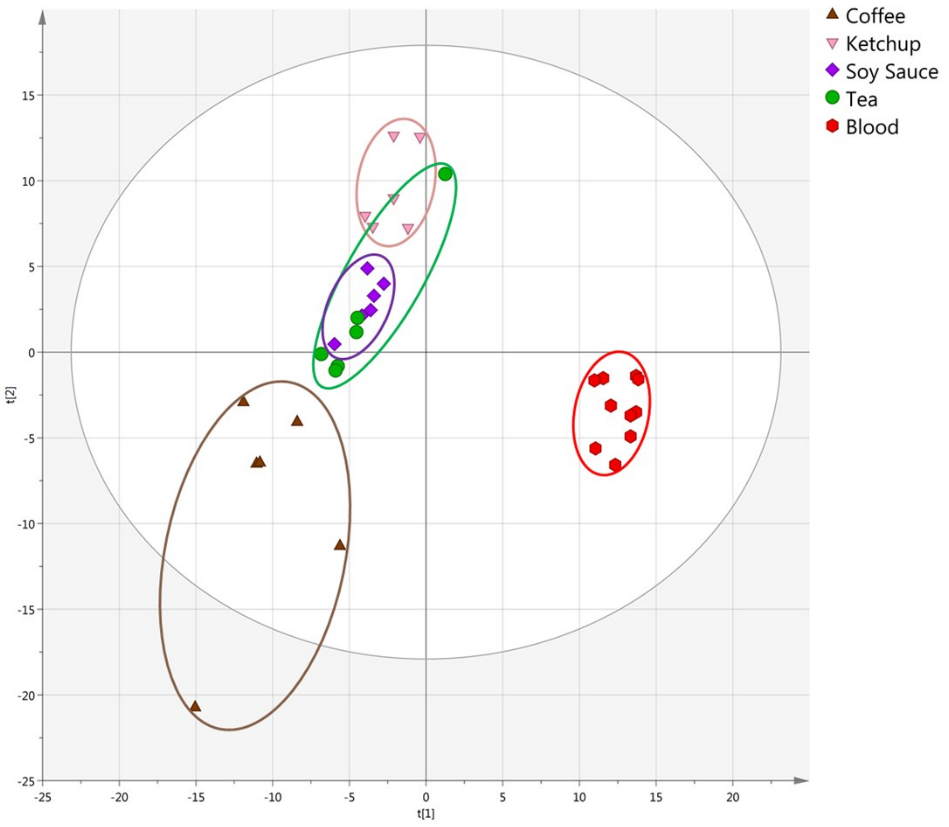


**Figure S6: PCA score plot of 2 month old blood and possible interferents.**

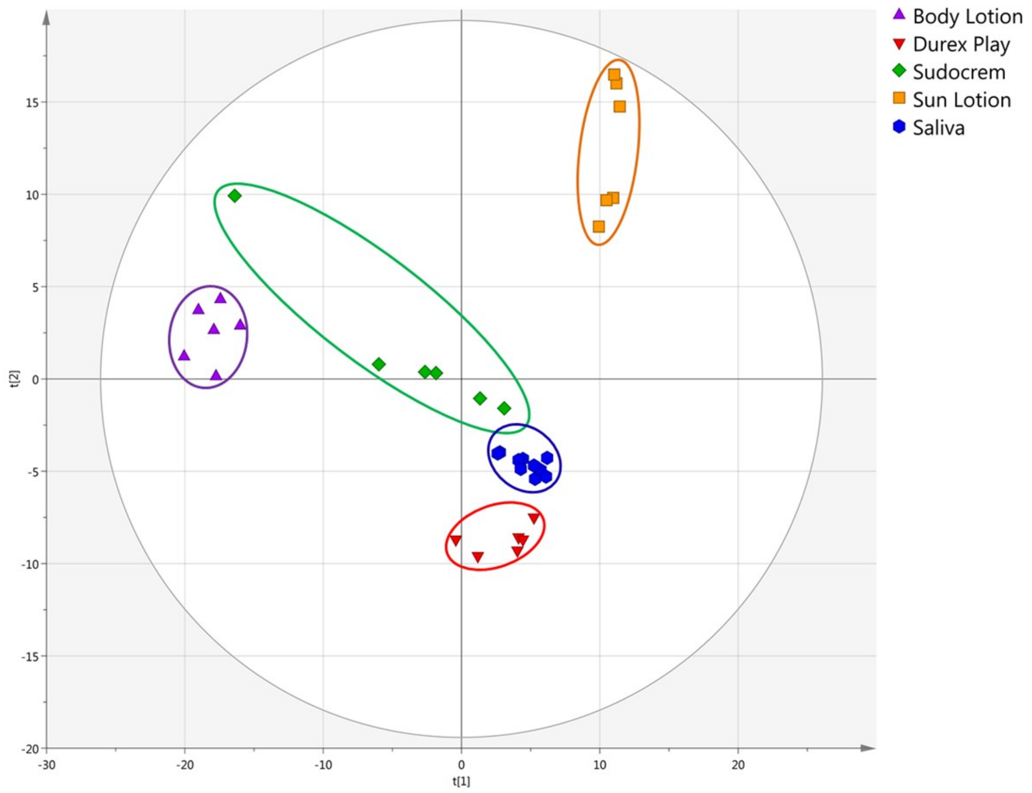


**Figure S7: PCA of 2 month old saliva and possible interferents.**

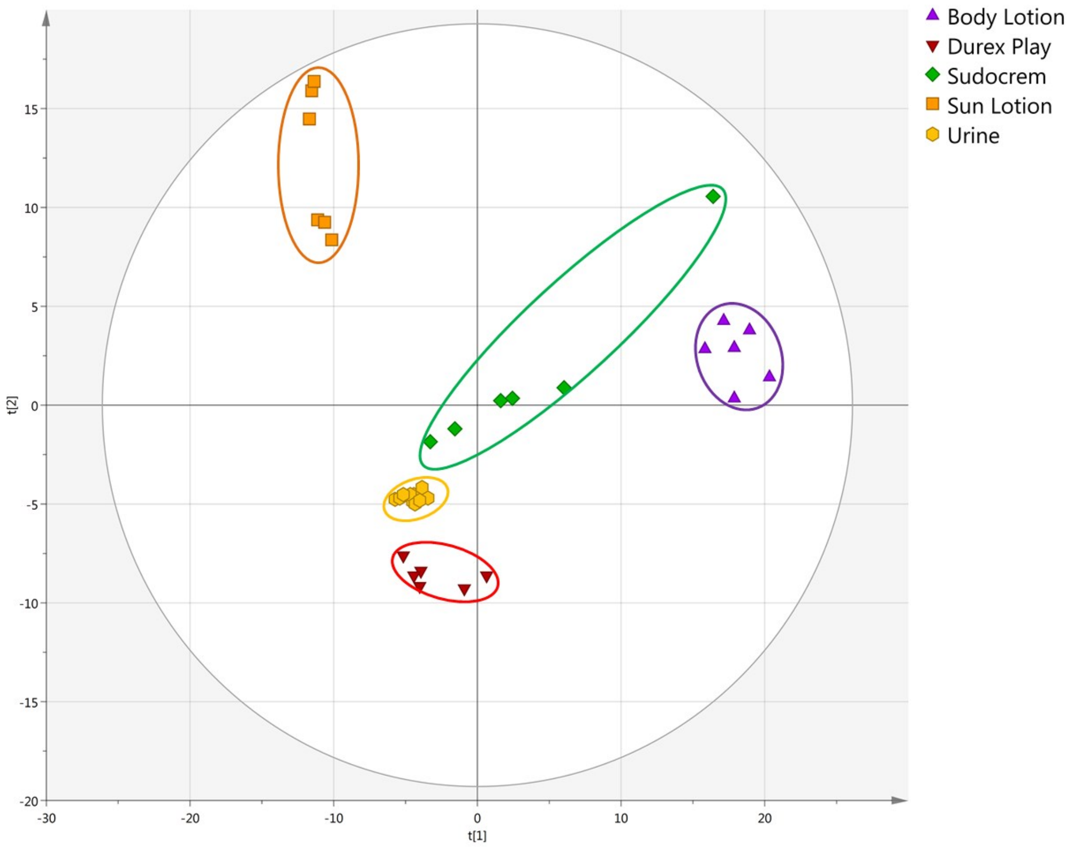


**Figure S8: PCA of 2 month of urine and possible interferents.**

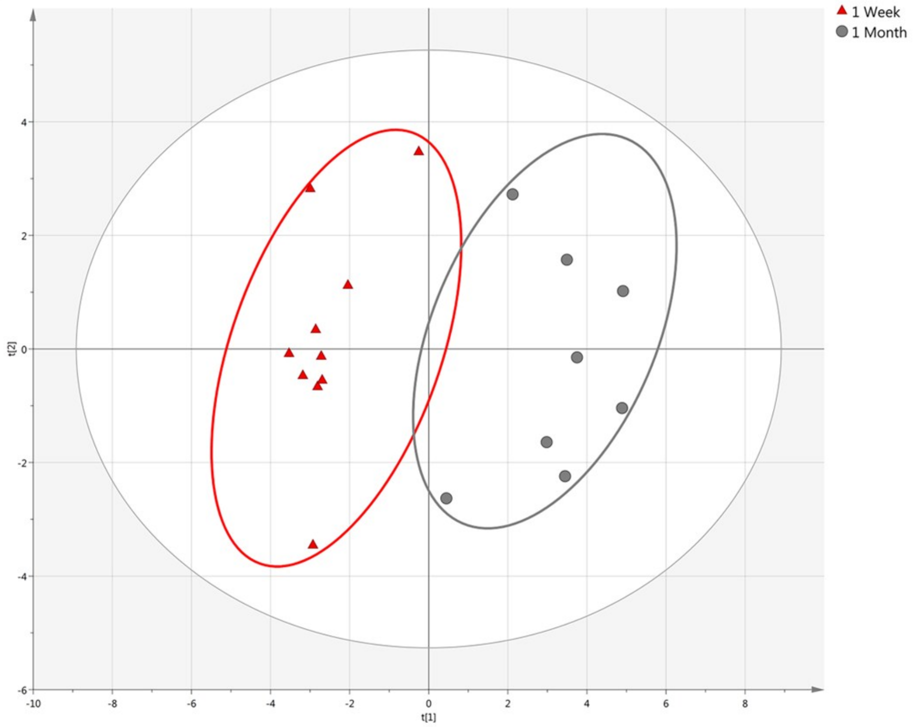


**Figure S9: PCA score plot of 1 week and 1 month blood samples.**

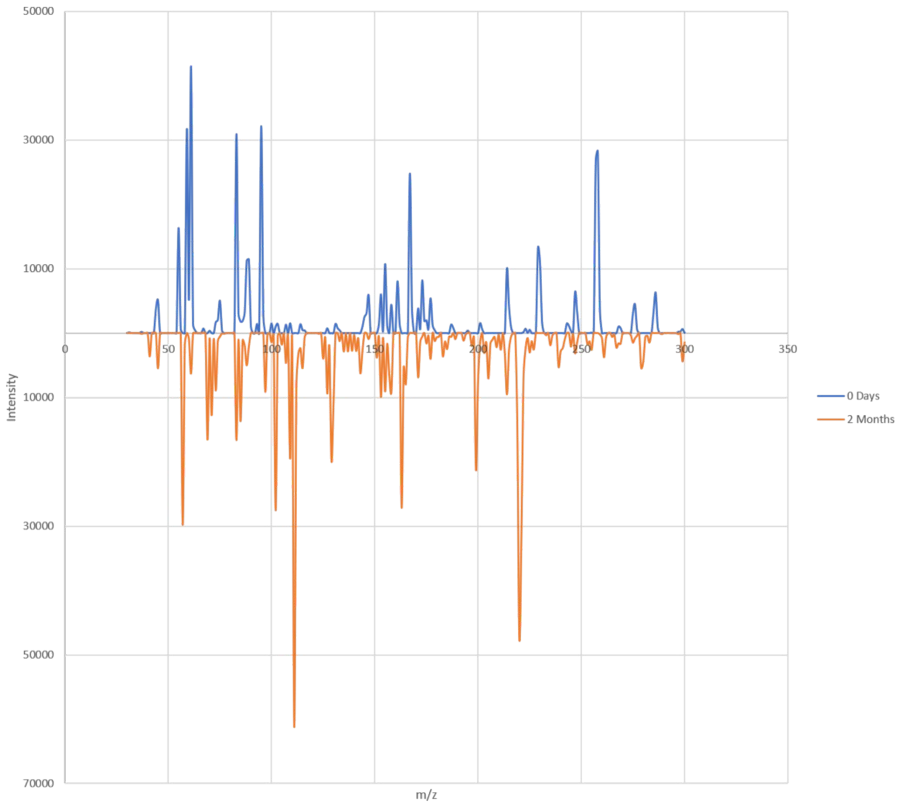


**Figure S10: Typical spectra obtained from unaged (top) and 2 month old (below) blood.**

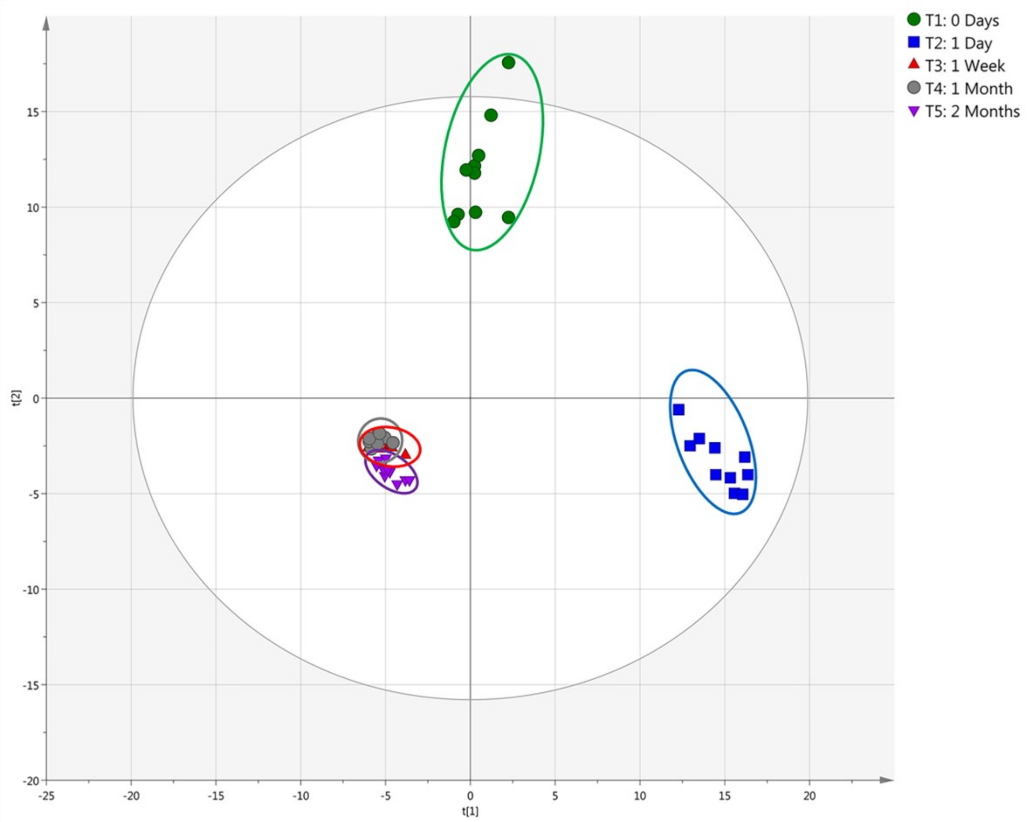


**Figure S11: PCA score plot of saliva aged 0 days to 2 months.**

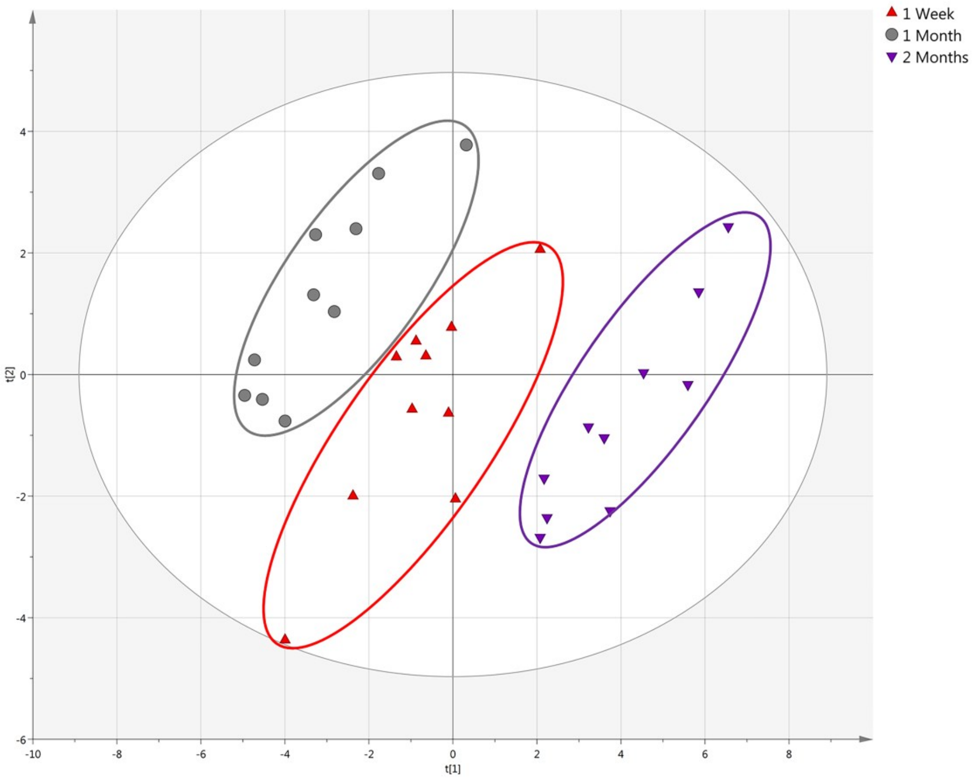


**Figure S12: PCA score plot of saliva aged 1 week, 1 month and 2 months.**

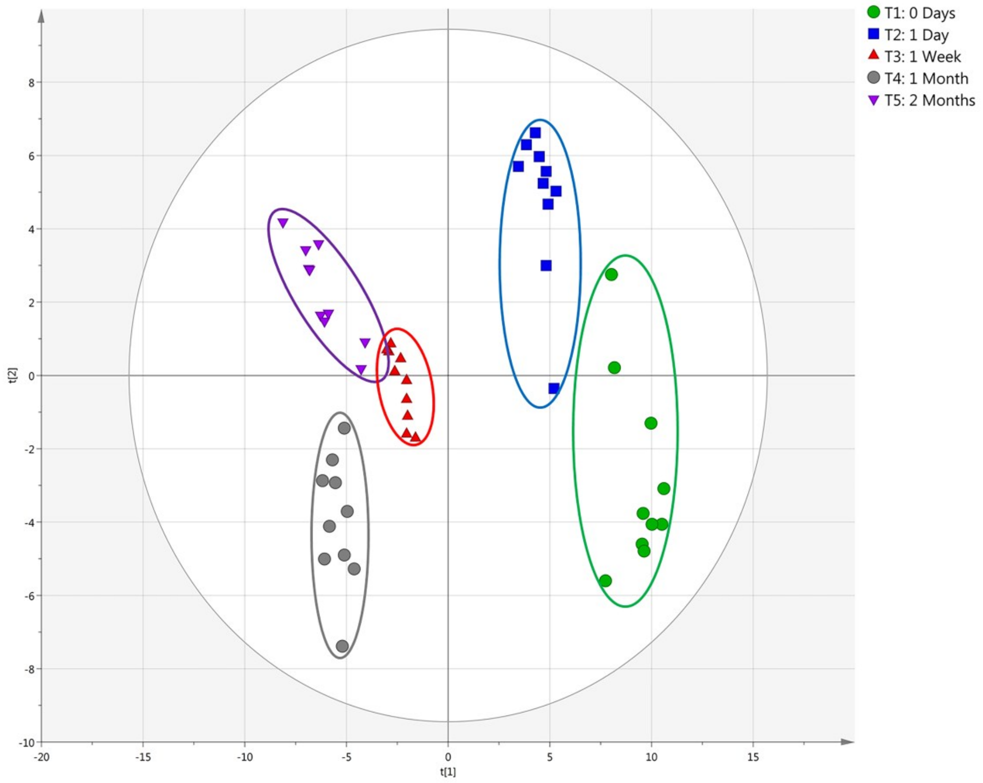


**Figure S13: PCA scores plot of urine aged 0 days to 2 months**

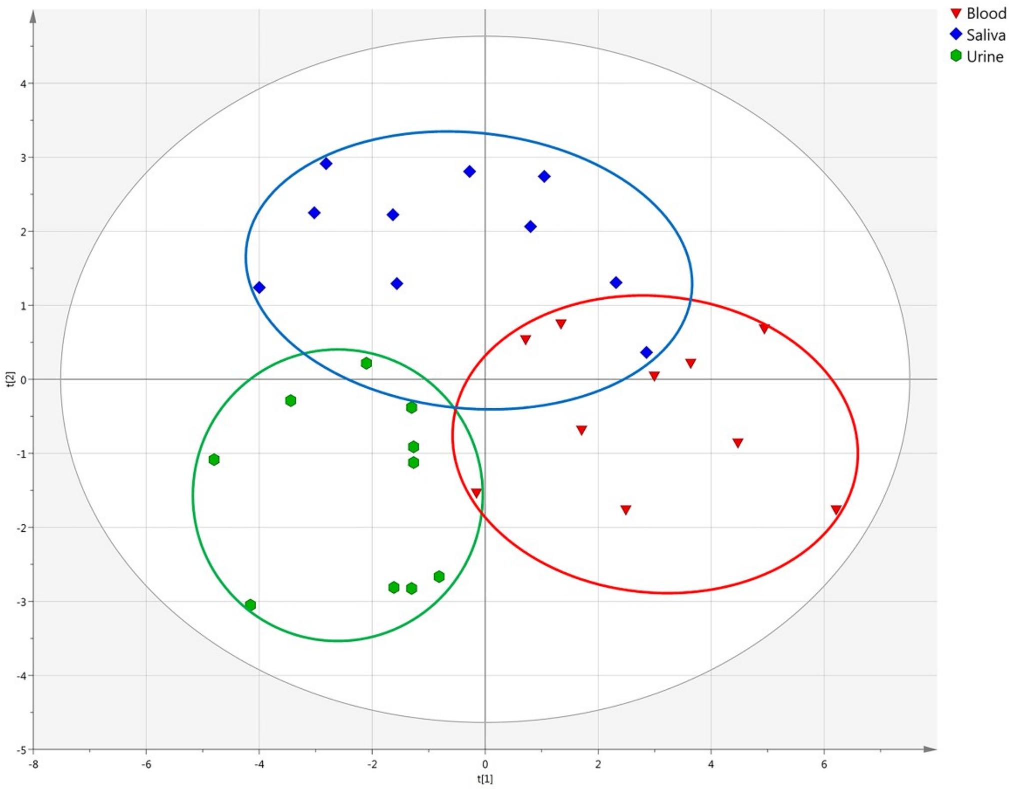


**Figure S14: PCA score plot of blood, saliva and urine aged 2 months.**

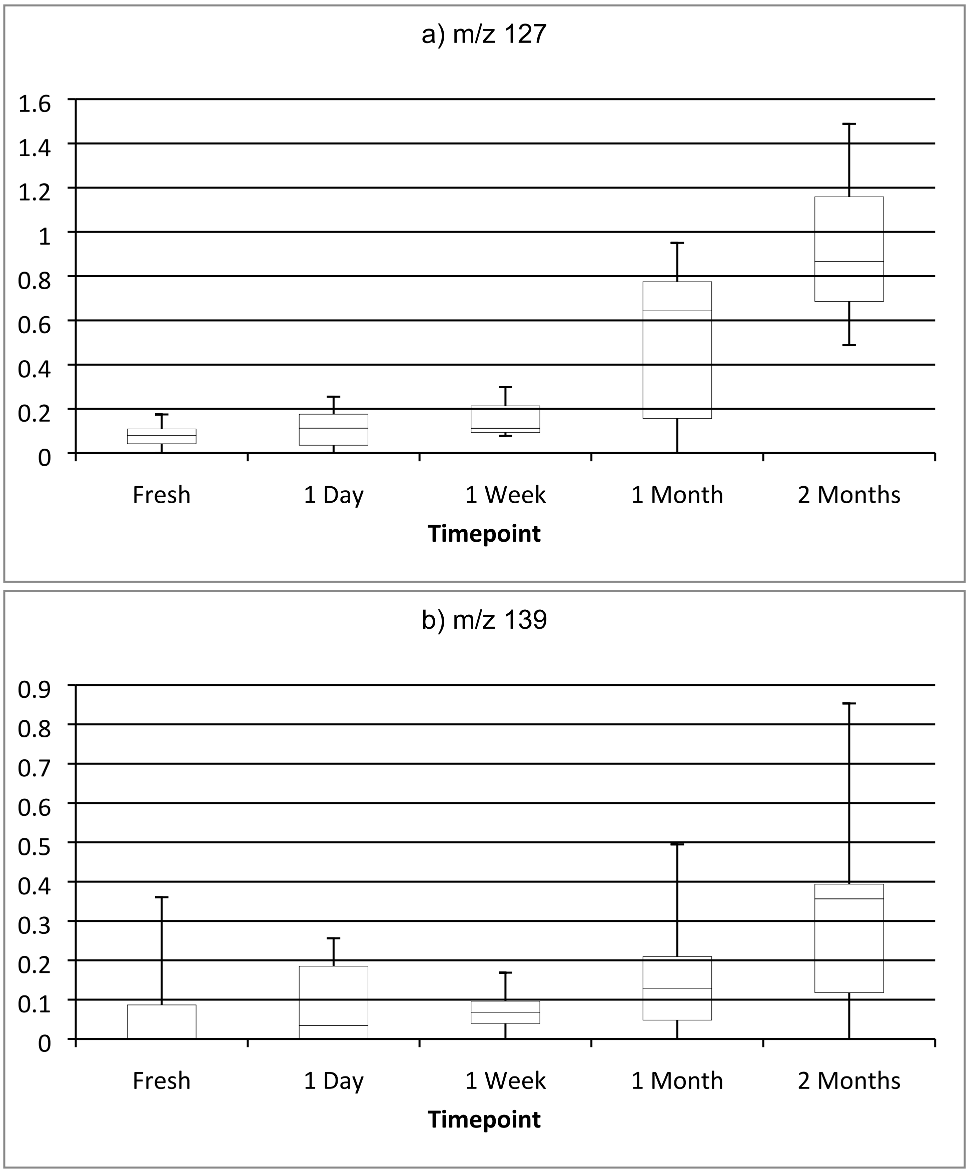


**Figure S15: Box plots of ion intensity increasing with age of blood sample, a) m/z 127 (6-methyl-5-hepten-2-one) and b) m/z 139 (2-pentylfuran).**

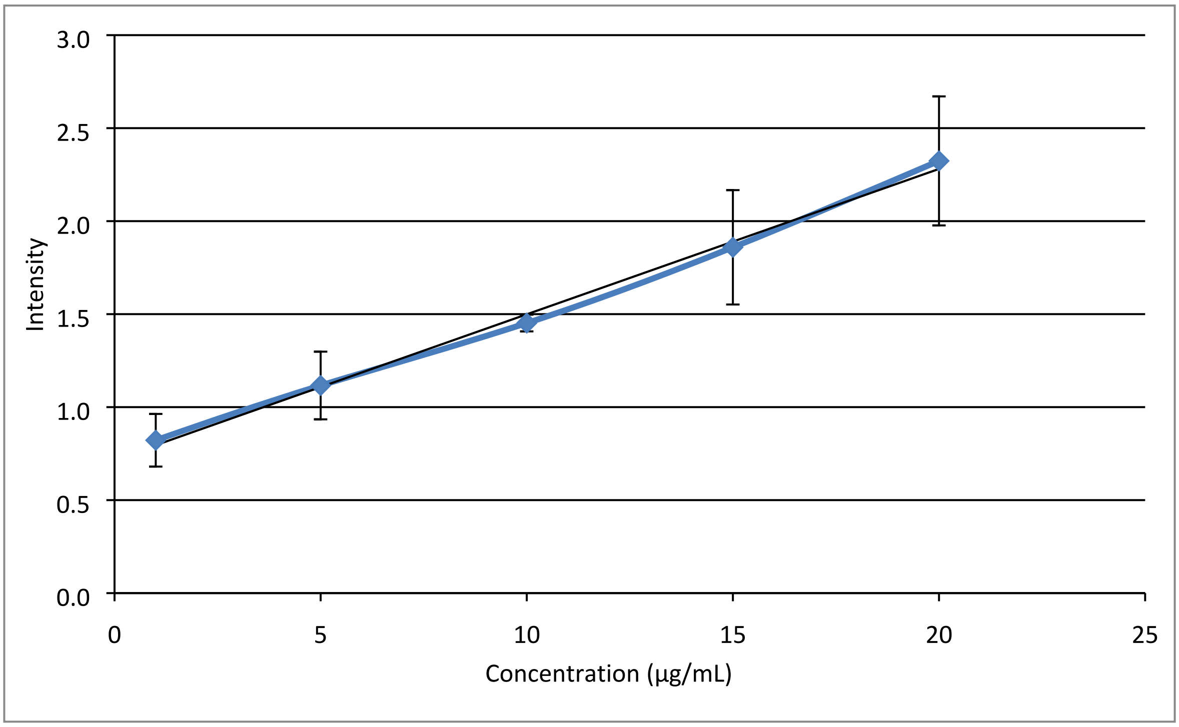


**Figure S16: Calibration curve for 3-methylbutanamide over a range of 1-20 µg/mL, based on 6 replicates of each concentration ($R^2$ of 0.9959 and average %RSD of 13%).**

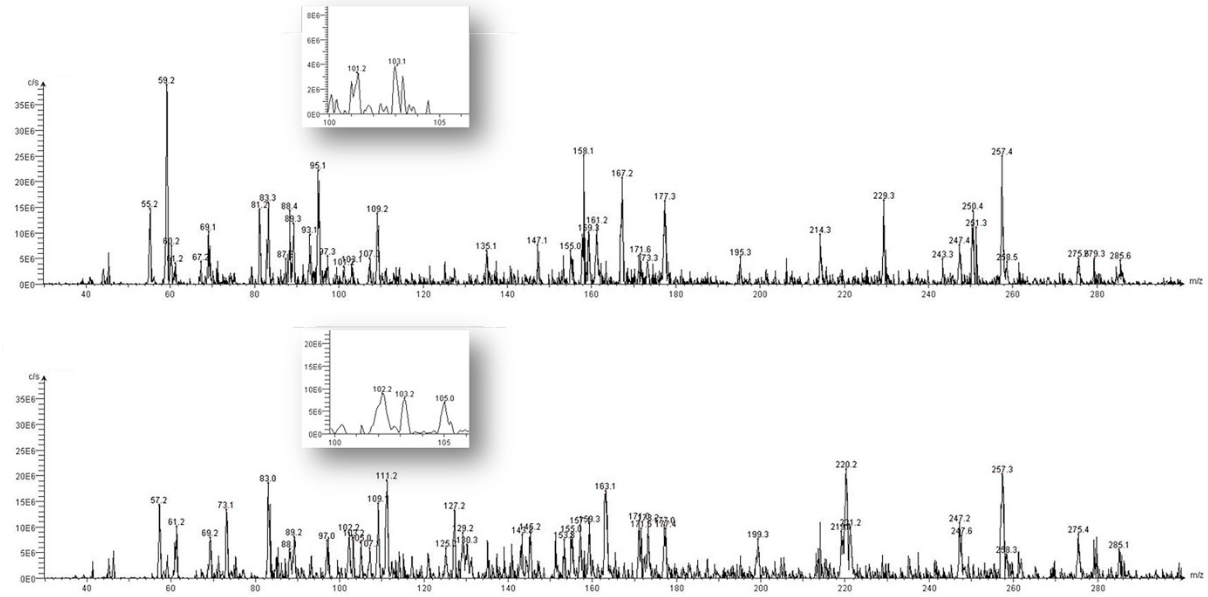

**Figure S17: Positive mode APCI mass spectra of blood aged 0 days (above) and blood aged 2 months (below).**
